# Interleukin-6 and its association with outcome in traumatic brain injury: a prospective cohort

**DOI:** 10.1186/s13049-025-01430-2

**Published:** 2025-07-26

**Authors:** Eder Cáceres, Afshin A. Divani, Juan Olivella-Gomez, Mario Di Napoli, Luis F. Reyes

**Affiliations:** 1 Bioscience PhD. School of Engineering, Universidad de La Sabana, Chía, Colombia; 2 Unisabana Center for Translational Science, School of Medicine, Universidad de La Sabana, Chía, Colombia; 3 Department of Critical Care, Clínica Universidad de La Sabana, Chía, Colombia; 4 Department of Neurology, University of New Mexico Health Sciences Center, Albuquerque, NM USA; 5 Neurological Service, Dell’Annunziata Hospital, Sulmona, L’Aquila Italy

**Keywords:** Traumatic brain injury, Trauma, Inflammation, Biomarker, Prognostication, Inflammatory response, Interleukin

## Abstract

**Background:**

Traumatic brain injury (TBI) continues to be a major cause of death and disability worldwide. Biomarkers for treatment and prognostication are needed for counseling and clinical management.

**Objective:**

In this study, we evaluated the ability of serum IL-6 to predict mortality and disability in a population whith moderate and severe TBI (msTBI).

**Methods:**

Adult patients with msTBI were included consecutively from December 2019 to August 2023. Clinical data were collected during hospital stays and functional outcome was established at 6 months using GOSE. Serum IL-6 levels were measured on day 0, day 3 and day 7 after injury.

**Results:**

Eighty-eight patients were recruited and completed 6-month follow-up. Clinical variables associated with the 6-month adverse outcome were admission GCS (OR 0.77 95% CI 0.67–0.87, *p* < 0.001), age (OR 1.10 95% CI 1.03–1.1, *p* = 0.001), Rotterdam score (OR 2.8 95% CI 1.7–5.0, *p* < 0.001), hospital infections (OR 4.7 95% CI 1.9–12.1, *p* < 0.001) and day-0 IL-6 (OR 1.1 95% CI 1.08–1.13, *p* < 0.001). When adjusted for age, severity of injury,and the presence of a hospital infection, day-0 IL-6 was significantly associated with the adverse outcome at 6 months (OR 1.15 95% CI 1.1–1.2, *p* = 0.031). Area under the curve (AUC) of 89% (95% CI 82%—96%). Calculated sensitivity and specificity were 75% and 89%, respectively, at a cut-off point of 59 pg/ml.

**Conclusion:**

In a population of msTBI, levels of serum interleukin-6 within the first 24 h after injury is an independent predictor of 6-month mortality and disability with a net benefit in clinical decision-making across relevant threshold probabilities.

## Background

Traumatic brain injury (TBI) is a leading cause of death and disability worldwide [1]. Survivors often face long-term functional limitations, hindering their social and work reintegration [2, 3]. An estimated 50 to 60 million people experience TBI annually, costing the global economy around US$400 billion, including acute care and rehabilitation [4]. The incidence of TBI is expected to rise with increasing population densities, aging, and motor vehicle use [1].

Predicting recovery after TBI is complex due to the injury's heterogeneity and individual responses [5, 6]. Even after surviving a moderate-severe TBI (msTBI) patients are at a higher risk of complications like seizures, infections, and dementia [7, 8]. Estimates based on the TBI model system (TBIMS National Database) in the US, that includes people 16 years of age and older who received inpatient rehabilitation services for a primary diagnosis of TBI, report that after five years, 22% are still homebound, and 22% have died [9]. Prognostication is common in TBI care, as families frequently seek guidance on expected outcomes or decisions regarding life-support withdrawal. Accurate predictions can help align care with patient preferences, but inaccurate predictions may lead to premature withdrawal of care in patients who could recover. Guidelines advise against using single clinical variables (e.g., age, Glasgow Coma Scale (GCS), head CT findings) as sole predictors of mortality or outcomes [10].

Clinical prediction models, like the CRASH and IMPACT models, have been developed to assess outcomes after TBI. The CRASH model predicts 14-day mortality and six-month severe disability using factors such as age, GCS, pupil status, extracranial injuries, and head CT findings [11]. An online calculator for high- and low-income settings is available (https://crash2.lshtm.ac.uk/Risk%20calculator/index.html). The IMPACT model (core and extended versions) adds variables like hypotension, hypoxia, Marshall CT classification, glucose, and hemoglobin to improve prognostication in msTBI [12]. Its online calculator is accessible at (http://tbi-impact.org/?p=impact/calc). These models are recommended for use in clinical practice, with an understanding of their limitations and patient preferences [10]. Inflammatory molecules, including interleukins and chemokines, link primary and secondary brain injury and are emerging as potential biomarkers for TBI prognosis [13, 14]. Identifying immune response dynamics post-injury helps define specific endophenotypes, guiding precision medicine and novel therapeutic targets in TBI [15]. TBI involves brain function disruption due to mechanical injury, typically from a direct impact on the brain or a rapid acceleration-deceleration [16]. The primary injury causes structural changes such as contusions, hemorrhages, and axonal injury [17], leading to cellular disruption, oxidative stress, and energy disturbances [18]. This disruption triggers the release of intracellular components that initiate an inflammatory response aimed at debris clearance and tissue repair [19]. Early activation of astrocytes and microglia in the brain releases cytokines, chemokines, and vascular adhesion molecules, attracting immune cells [20]. The type and severity of injury, age, and premorbid status influence the immune response. A balanced inflammatory response is essential for optimal recovery, but dysregulation can prolong recovery and have lasting effects [20, 21].

Among inflammatory molecules, IL-6 plays a central role. Produced by microglia, neurons, and endothelial cells, IL-6 has both inflammatory and regenerative functions [22]; its levels rise early in the brain post-injury, supporting the concept of local inflammation before peripheral immune cell recruitment [23, 24]. IL-6 promotes microglia activation and local inflammation while enhancing cell migration, synaptic activity, and vascularization, contributing to recovery [25–27]. IL-6 knockout models show impaired immune response, increased oxidative stress, and slower recovery [28]. IL-6 also regulates the transition from innate to adaptive immunity by modulating neutrophil inhibition and recruiting monocytes and T cells [29]. Dysregulation of this process may lead to chronic disease progression [30].

In the TRACK-TBI cohort, IL-6 and other biomarkers (e.g., IL-15, CRP, serum amyloid protein) could differentiate mild from moderate-to-severe TBI, with IL-6 associated with structural damage on head CT [31]. A systematic review found mixed evidence for IL-6 as a prognostic biomarker, with some studies showing associations with poorer outcomes and others showing no significant correlation [32]. Standardization of IL-6 measurement and interpretation, as well as further research on its prognostic value, are needed [10].

While IL-6 shows potential as a prognostic biomarker in TBI, its clinical utility remains unclear. In this study, we assess the association of IL-6 levels with mortality and disability in a prospective cohort of msTBI.

## Materials and methods

This study was approved by the Institutional Review Board (IRB) under local regulations and the Declaration of Helsinki, with informed consent obtained from patient representatives. All clinical data were anonymized and collected using REDCap, a secure electronic data capture system provided by Universidad de La Sabana.

### Population

We included patients from a single trauma center in Chía, Colombia, between December 2019 and August 2023. Inclusion criteria were age > 17, moderate or severe TBI within 24 h of trauma, and a hospital stay of at least 48 h in the Neuro-Intensive Care Unit (Neuro-ICU). Patients with a modified Rankin Scale (mRS) > 2 due to pre-existing disability or admitted after 24 h post-injury were excluded.

### Definitions

After clinical resuscitation and stabilization of vital signs, the severity of TBI was classified according to the GCS. If sedation was administered, the last documented GCS prior to sedation was used.

Brain injury severity on head CT was assessed using the Marshall classification and the Rotterdam score, both of which have been validated for correlation with TBI outcomes [33–38].

The Injury Severity Score (ISS) was used to assess the severity of non-head injuries. An ISS > 15 or multiple body regions with an Abbreviated Injury Scale (AIS) > 3 were considered indicators of major trauma [39].

The International Mission for Prognosis and Analysis of Clinical Trials in TBI (IMPACT) lab model was used to predict mortality and disability at 6 months. This model includes age, motor GCS score, pupillary reactivity, CT findings, and admission hemoglobin and glucose levels [12, 40].

Functional outcomes were measured using the Glasgow Outcome Scale-Extended (GOSE) via a standardized phone interview at 6 months. Outcomes were dichotomized into adverse (GOSE < 4) or favorable (GOSE > 4) [31, 41–43].

Infectious complications were defined according to the Infectious Disease Society of America/American Thoracic Society guidelines definitions, including Ventilator-associated Pneumonia (VAP), Ventilator-associated Tracheitis (VAT), Catheter-Associated Urinary Tract Infection (CAUTI), Surgical Site Infection, and Catheter-Related Bloodstream Infection [44–47].

### Data collection

Blood samples were taken on admission (within 24 h), day 3, and day 7 post-injury. Serum IL-6 levels were measured using the MILLIPLEX® Luminex immunoassay system (Merck KGaA, Darmstadt, Germany). Blood samples were collected, and the plasma was transferred into 2 to 3 polypropylene tubes and stored at −80 °C until analysis. The assay sensitivity or minimum detectable concentration for IL-6 with the overnight protocol used in our analysis is 0.14 pg/ml.

Demographics, severity, infection, hospital stay, and 6-month outcomes were recorded prospectively from the electronic medical records. Data management was handled using REDCap, which supports secure, web-based data capture and export for statistical analysis.

### Statistical analysis

Continuous variables were summarized as mean ± standard deviation (SD) for normally distributed data and median with interquartile range (IQR) for non-normally distributed data, with normality assessed using the Shapiro–Wilk test. Categorical variables were presented as counts and percentages. Group differences were evaluated using chi-square or Fisher’s exact test for categorical variables and Student’s t-test or Mann–Whitney U test for continuous variables, based on the distribution.

We constructed a multivariate logistic regression model to investigate the association between IL-6 levels at admission, day 3, and day 7 with 6-month mortality and functional outcomes,. Variables with a *P*-value < 0.10 in univariate analysis were included in the model. Stepwise selection (forward and backward) and LASSO regularization were applied to mitigate multicollinearity and prevent overfitting, improving model robustness.

Logistic regression results were reported as odds ratios (OR) with 95% confidence intervals (CI). Model discrimination was assessed using the area under the receiver operating characteristic (ROC) curve (AUC), with higher values indicating better predictive accuracy. Calibration was tested using the Hosmer–Lemeshow goodness-of-fit test, ensuring model-predicted probabilities align with observed outcomes.

In addition, we evaluated the predictive ability of IL-6 levels as continuous and dichotomized (using optimal cut-offs identified via Youden’s Index) by comparing them against the IMPACT model’s predictions for adverse outcomes (GOSE ≤ 4). We applied DeLong’s test, to formally compare the predictive performance between IL-6 and the IMPACT score. Comparative AUC values were also computed to assess whether IL-6 adds incremental value to the IMPACT model. This comparison is to evaluate whether IL-6 alone or in conjunction with the IMPACT model changes predictive accuracy.

To assess IL-6's clinical utility as a prognostic marker, decision curve analysis (DCA) was performed. DCA compares the net benefit of using IL-6 at various threshold probabilities with the strategies of treating all or no patients, aiding in assessing IL-6's impact on clinical decision-making.

Missing data were handled through multiple imputations under the assumption of data missing at random (MAR). Sensitivity analyses, including complete-case analysis, were performed to confirm result robustness, comparing imputed versus non-imputed datasets.

Internal validation of the logistic regression model was conducted using bootstrap resampling with 1,000 iterations to evaluate the stability of performance metrics, including AUC and calibration slope. All tests were two-tailed, with *P*-values < 0.05 considered statistically significant.

Analyses were conducted using R Studio (Version 2023.09.1 + 495). The following packages were employed: `glmnet` for LASSO regularization, `pROC` for ROC analysis, `rmda` for decision curve analysis, and `rms` for regression modeling and calibration.

## Results

### Population

We collected data on 88 patients admitted to the ICU with TBI, 79% (70/88) male, with a mean age of 41 ± 19 years. The leading causes of trauma were road traffic accidents (53%), falls (25%), cycling accidents (13%), violence (5%), and other causes (4%). At admission, the GCS indicated moderate TBI in 50% and severe TBI in 50%, with a median GCS of 8 (IQR 6–12). The median Injury Severity Score (ISS) was 26 (IQR 17–34). Other significant injuries (AIS > 3) included thoracic trauma (39%), limb injuries (28%), and abdominal trauma (16%).On CT, the median Rotterdam score was 2 (IQR 2–3). Marshall classification revealed Diffuse Injury I (27%), Diffuse Injury II (43%), Diffuse Injury III (10%), and evacuated mass lesions (20%). Common CT findings included epidural hematomas (62%), contusions (59%), traumatic subarachnoid hemorrhages (51%), and subdural hematomas (30%). Laboratory results, including blood tests and arterial blood gases (ABG), are summarized in Table 1.

**Table 1:** Demographic characteristics of moderate-severe TBI patients

	Overall (*n* = 88)
Age, mean ± SD	41 ± 19
Sex, male % (n)	79 (70)
Causes of trauma % (n)	
Motor vehicle accident	53 (47)
Falls	25 (22)
Cycling	13 (11)
Violence	5 (4)
Severity of TBI by GCS % (n)	
Moderate	50 (44)
Severe	50 (44)
Admission GCS, median (IQR)	8 (6–12)
AIS head median (IQR)	4 (3–4)
Extracranial injuries % (n)	
Thorax	39 (35)
Limbs	28 (25)
Abdomen	16 (14)
Apache II, mean (SD)	14 (7)
IMPACT probability of disability %, median (IQR)	29 (13–33)
Charlson, mean (SD)	0 (0)
Rotterdam score, median (IQR)	2 (2–3)
Marshall CT classification % (n)	
Diffuse Injury I	27 (24)
Diffuse Injury II	43 (38)
Diffuse Injury III	10 (9)
Evacuated mass lesion	20 (17)
Primary injury on CT % (n)	
Epidural hematoma	62 (55)
Contusion	59 (52)
tSAH	51 (45)
Subdural hematoma	30 (27)
**Lab tests on admission**	
WBC, median (IQR) × 10^3^/dl	16.0 (12–20)
Neut %, median (IQR)	82 (75–88)
Hg grs/dl, median (IQR)	14.1 (12.35–15.1)
Platelet count, mean ± SD × 10^3^/ul	230.4 (81)
Serum glucose, median (IQR), grs/dl	130 (110–162)
Creatinine, median (IQR), mg/dl	0.9 (0.7–1.1)
Sodium, median (IQR), mEq/L	140 (137–142)
pH on ABG, median (IQR)	7.34 (7.2–7.41)
PaO2, median (IQR), mmHg	88 (71–126)
PaCO2, median (IQR), mmHg	35 (30–41)
ABG bicarbonate, mean (SD), mmHg	18.8 (3.5)
Lactate, mean (IQR), mmol/L	2.6 (1–7-3.7)
**Hospitalization**	
ICU LOS, median (IQR), days	6 (4–15)
Hospital LOS, median (IQR), days	11 (7–29)

*GCS *Glasgow Coma Scale, Moderate TBI: GCS 9–12. Severe TBI: GCS 3–8. *CT *computed tomography scan of the head, *IMPACT *International Mission for prognosis and analysis of clinical trials in TBI, *tSAH *traumatic Subarachnoid Hemorrhage, *WBC *white blood cell count, *Neut *neutrophil, *Hg *hemoglobin, *ABG *arterial blood gases, *ISS *Injury Severity Score, *LOS *Length of stay, *GOSE *Glasgow Outcome Scale-Extended

### ICU course

On admission, 71% of patients [48] required mechanical ventilation for a median duration of 6 days (IQR 3–12). During ICU stay, 46% [41] of patients developed infections, most commonly around day 5 (IQR 4–7). Respiratory tract infections occurred in 36% (32 patients), including ventilator-associated pneumonia (9 cases) and ventilator-associated tracheitis (23 cases). Other infections included catheter-associated urinary tract infections (6%) and bloodstream infections (5%). The infection diagnosis was made after 5 days (4–7) in hospital.

**Fig. 1:**
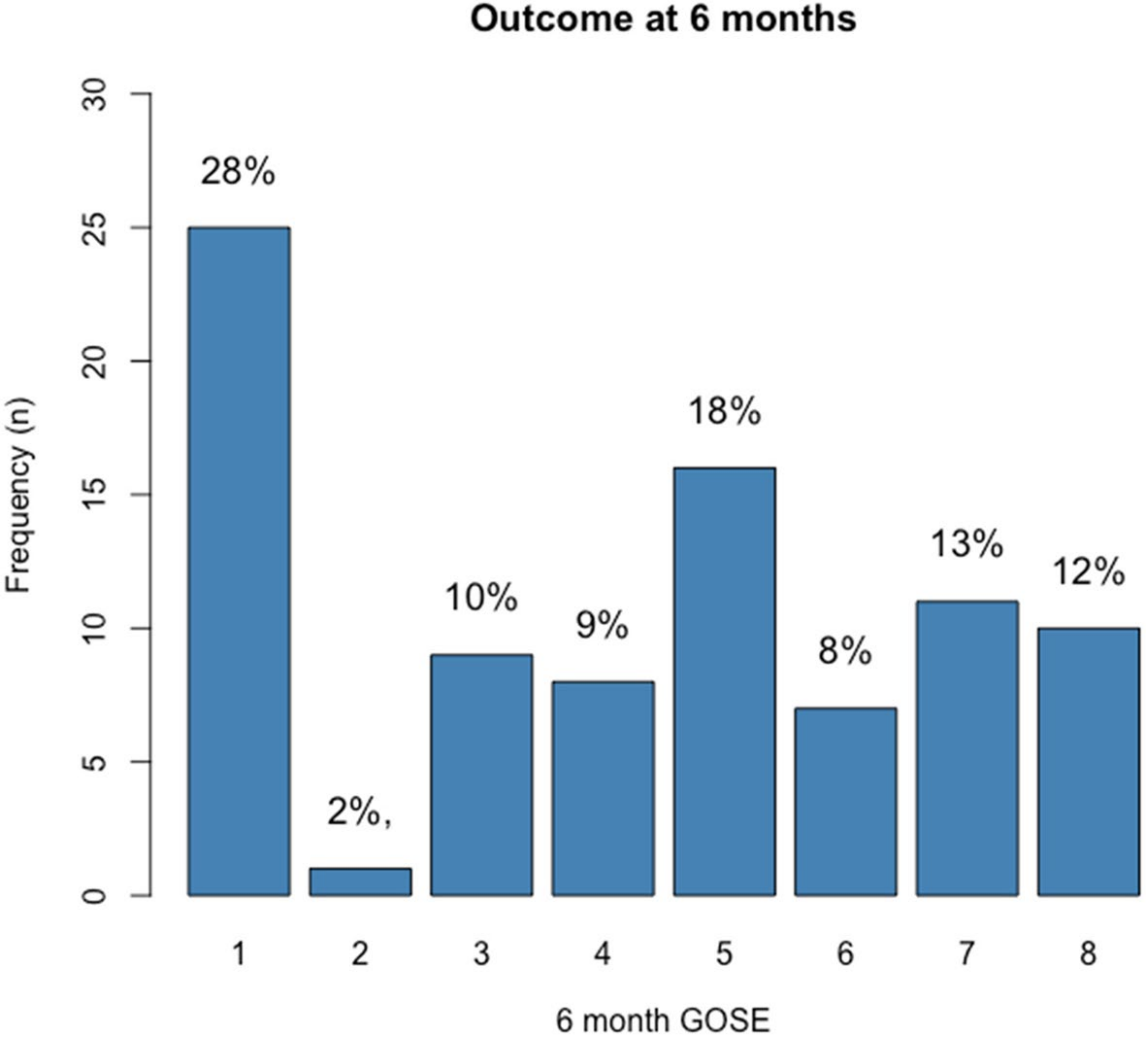
Functional outcome at 6 months in the entire cohort. Six months after TBI, the outcome of the 88 surviving patients was assessed using the GOSE. The percentage by category has been placed on top of each bar

The infection rate was significantly higher in patients with adverse outcomes (GOSE ≤ 4) at 6 months compared to those with favorable outcomes (60% vs 24%, *P* = 0.0013). ICU length of stay (LOS) was a median of 6 days (IQR 4–15), and hospital LOS was 11 days (IQR 7–29). Hospital mortality was 16%, while 6-month mortality was 28%. Functional outcomes are shown in Fig. 1.

### IL-6 analysis

IL-6 measurements were available for all 88 patients on day 0, 76 on day 3, and 52 on day 7. Missing samples were due to patient deaths (6 before day 3, 14 before day 7), transfers, or lack of valid results. Median IL-6 levels were day 0, 44.11 pg/ml (IQR 15.68–87.7); day 3, 14.81 pg/ml (IQR 5.94–39.9); and day 7, 12.11 pg/ml (IQR 5.5–24.68). A significant decrease in IL-6 levels was observed across time points (*P* < 0.001) (Fig. 1a).

Patients with hospital infections had significantly higher IL-6 levels at all time points (day 0: 51.98 pg/ml vs. 19.44 pg/ml, *P* < 0.001; day 3: 28.67 pg/ml vs 7.77 pg/ml, *P* < 0.001; day 7: 19.54 pg/ml vs. 5.71 pg/ml, *P* = 0.0011). Higher day 3 IL-6 was also associated with hospital mortality (*P* = 0.02), although no differences were found for IL-6 on day 0 or day 7.

### Functional and mortality outcomes

**Fig. 2:**
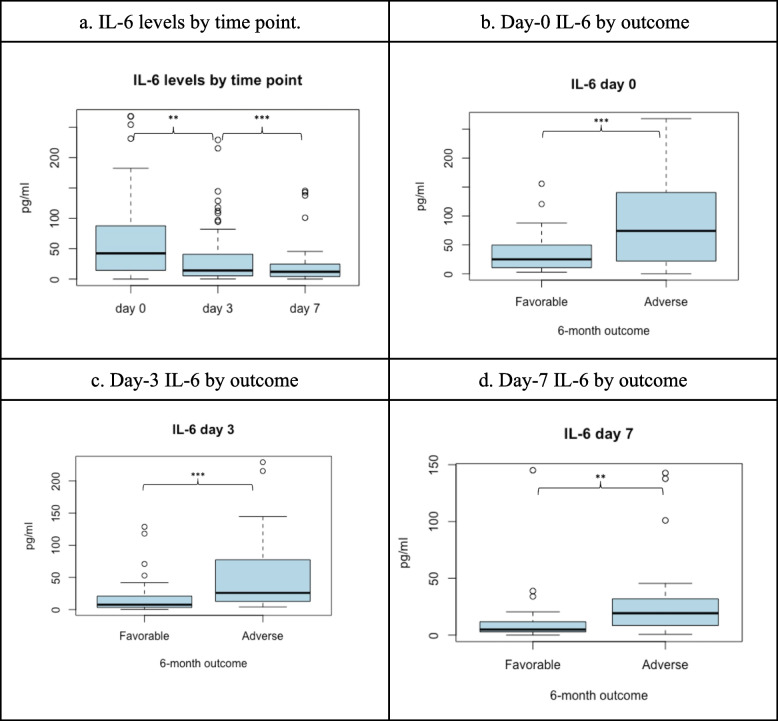
IL-6 levels according to time points and favorable versus adverse outcome

Patients with a 6-month adverse outcome (GOSE ≤ 4) had significantly higher IL-6 levels on day 0 (*P* < 0.001), day 3 (*P* < 0.001), and day 7 (*P* = 0.0013) (Fig. 1b, c, d). IL-6 levels on day 0 and day 3 were also higher in those who died by 6 months (*P* = 0.007 and *P* = 0.02, respectively). A trend towards higher day 7 IL-6 in non-survivors was observed but was not statistically significant (*P* = 0.051) (Fig. 2).

### Bivariate analysis

Factors associated with 6-month adverse outcomes included admission GCS (OR 0.77, 95% CI 0.67–0.87, *P* < 0.001), age (OR 1.10, 95% CI 1.03–1.1, *P* = 0.001), IMPACT-TBI probability of disability (OR 8.5 × 10^3^, 95% CI 308–5.7 × 10^5^, *P* < 0.001), Rotterdam score (OR 2.8, 95% CI 1.7–5.0, *P* < 0.001), hospital infections (OR 4.7, 95% CI 1.9–12.1, *P* < 0.001), and day 0 IL-6 (OR 1.1, 95% CI 1.08–1.13, *P* < 0.001) (Table 2).

**Table 2:** Bivariate analysis 6-month adverse outcome

Variable	OR (95% IC)	*P* value
Age	1.10 (1.03–1.12)	**0.001**
GCS on admission	0.77 (1.67–0.87)	** < 0.001**
Rotterdam score	2.8 (1.7–5.0)	** < 0.001**
IMPAC-TBI disability	8.5 × 103 (308–5.7 × 105)	** < 0.001**
Hospital infection	4.7 (1.9–12.1)	** < 0.001**
Day-0 IL-6	1.1 (1.08–1.13)	** < 0.001**

Regarding IL-6, day 0 IL-6 was negatively associated with GCS (*P* = 0.0005) but showed no association on day 3 or day 7 (*P* = 0.1 and *P* = 0.7, respectively). Day 7 IL-6 was positively associated with ISS (*P* < 0.001), while no association was found on day 0 or day 3 (*P* = 0.1, *P* = 0.5). The Rotterdam score was significantly associated with day 0 IL-6 (*P* < 0.001). IL-6 on day 0 also correlated with the IMPACT-TBI model’s predicted probability of adverse outcomes (*P* = 0.0017). Both day 0 and day 7 IL-6 levels were positively associated with hospital infections (*P* = 0.005 and *P* = 0.01, respectively), while day 3 was not (*P* = 0.5).

### Multivariate analysis

In a multivariate model adjusted for age, injury severity, and hospital infections, day-0 IL-6 was independently associated with 6-month adverse outcomes (OR 1.15, 95% CI 1.1–1.2, *P* = 0.031). Stepwise selection methods (forward and backward) and LASSO regularization were used to manage multicollinearity and prevent overfitting. LASSO model resulted in an optimal lambda (λ) of 0.017. Coefficients (Β) using the optimal λ were calculated for the variables of the model: age (B = 0.05, SE: 0.01), admission GCS (B = −0.29, SE: 0.07), infection (B = 0.38, SE:0.55) and day-0 IL-6 (B = 0.05, SE = 0.005). All variables were retained in the model and had an impact on the outcome according to their coefficient. The Hosmer–Lemeshow goodness-of-fit test confirmed adequate model calibration (*P* = 0.16) (Table 3). Day 3 and day 7 IL-6 levels were not significantly associated with the 6-month outcome after adjustment for age and severity (day 3: OR 1.0, 95% CI 0.9–1.0, *P* = 0.3; day 7: OR 1.0, 95% CI 0.9–1.0, *P* = 0.6).

**Table 3:** Multivariate analysis admission IL-6 and 6-month adverse outcome

Variable	OR (95% CI)	*P* value
Age	1.1 (1.1–1.12)	** < 0.001**
Admission GCS	0.7 (0.51–0.9)	** < 0.004**
Hospital infection	2.7 (0.7–10.7)	0.13
Day-0 IL-6	1.15 (1.12–1.21)	**0.031**

*Abbreviations GCS* Glasgow coma scale

### ROC and net benefit analysis using IL-6 as a biomarker

**Fig. 3:**
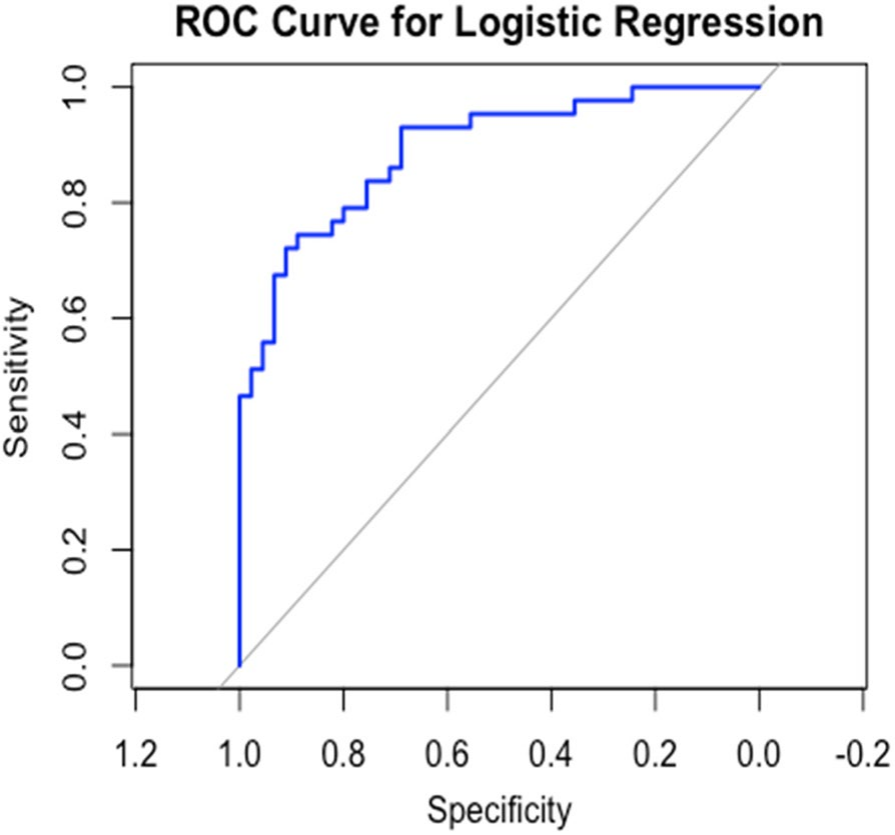
Receiver Operating Characteristic (ROC) Curve for IL-6 model Predicting Adverse Outcomes. This ROC curve illustrates the diagnostic performance of day-0 IL-6 levels in predicting adverse outcomes in patients with traumatic brain injury (TBI). The blue line represents the true positive rate (sensitivity) plotted against the false positive rate (1-specificity) across a range of IL-6 thresholds. The area under the curve (AUC) is a measure of the test’s overall accuracy, with higher values indicating better discriminative ability. In this analysis, IL-6 achieved an AUC of approximately 0.89, suggesting strong predictive power for identifying patients at risk of adverse outcomes. The diagonal gray line represents a reference for random chance (AUC = 0.5), highlighting the improvement provided by IL-6 levels over a non-informative model

**Fig. 4:**
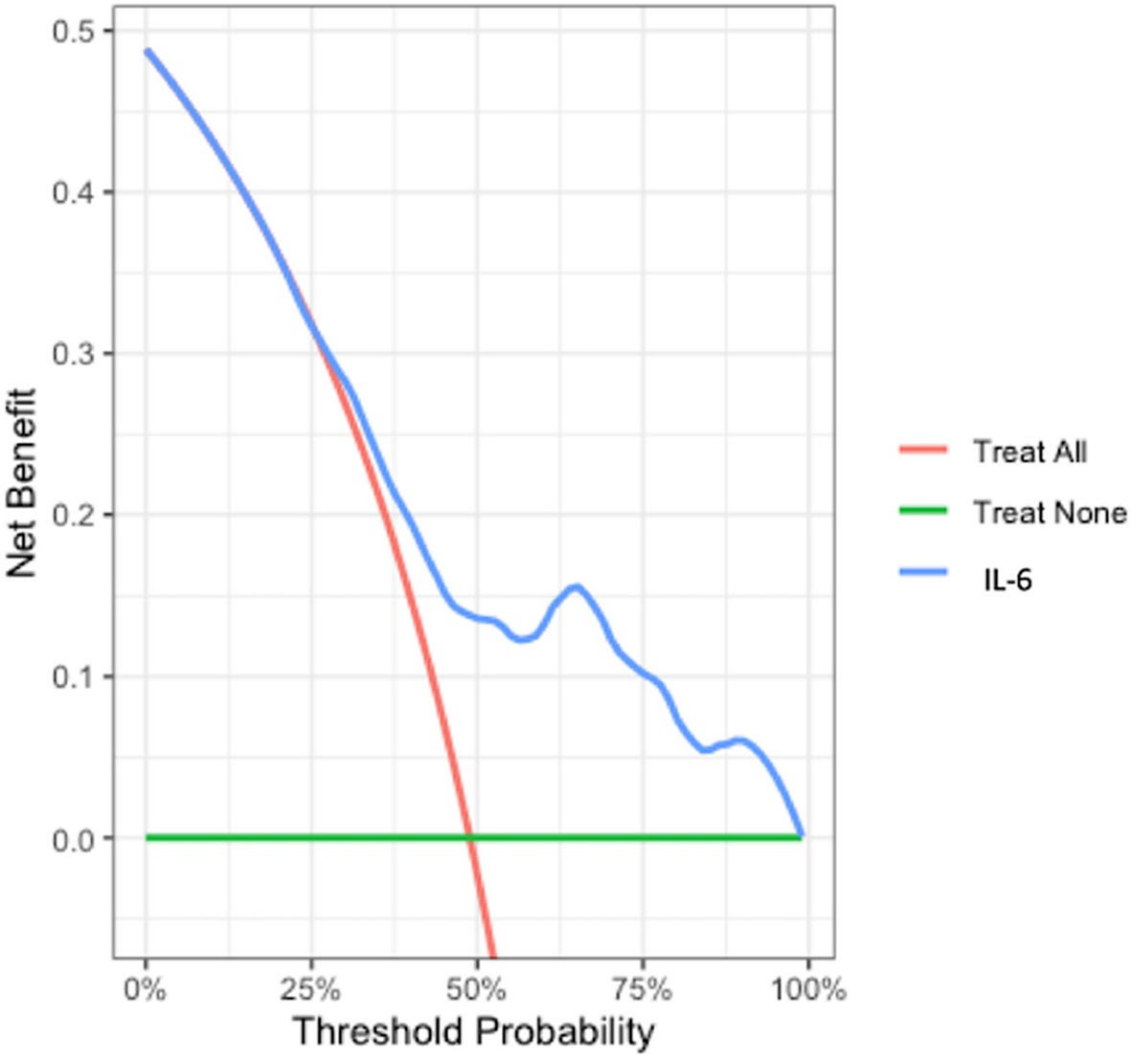
Decision Curve Analysis (DCA) for IL-6 as a Prognostic Biomarker in Traumatic Brain Injury (TBI). This Decision Curve Analysis (DCA) plot evaluates the clinical utility of IL-6 levels on admission for predicting adverse outcomes in TBI patients. The x-axis represents the threshold probability, or the probability above which a clinician might consider intervening based on predicted poor outcomes. The y-axis shows the net benefit, which is calculated by weighing the true positives against the false positives across different threshold probabilities. The green line (“Treat None”) represents the net benefit of not treating any patients for an adverse outcome, while the red line (“Treat All”) represents the net benefit of treating all patients for a potential adverse outcome. The blue line represents the net benefit of using IL-6 levels as a predictive biomarker. At threshold probabilities ranging from 0% to approximately 75%, the IL-6 model (blue line) demonstrates a higher net benefit compared to both the “Treat All” and “Treat None” strategies, suggesting that using IL-6 as a prognostic marker could help guide clinical decisions by identifying patients more likely to experience poor outcomes. This is particularly relevant for cases where clinicians are considering escalation of care or providing prognostic information to families. The observed net benefit of IL-6 in this range indicates its potential clinical value in distinguishing between patients who might benefit from early interventions and those who may not

ROC analysis for the ability of a model including age, GCS and day 0 IL-6 predicting adverse outcomes yielded an area under the curve (AUC) of 0.89 (95% CI 0.82–0.96) (Fig. 3). The optimal cut-off point of 59 pg/ml was determined using Youden’s Index, providing a sensitivity of 78% and specificity of 81%, positive predictive value (PPV) 79% and negative predictive value (NPV) 81%. To better evaluate the clinical utility of IL-6 as a prognostic biomarker, Decision Curve Analysis (DCA) was employed. DCA assessed the net clinical benefit of using IL-6 in decision-making and demonstrated its utility in predicting TBI outcomes beyond just the AUC values. The DCA plot illustrates the net benefit of using IL-6 for predicting an adverse outcome compared to using it in all or no patients. The model demonstrates a net benefit across a wide range of threshold probabilities, particularly for patients in whom the clinician is concerned about a poor prognosis. This suggests that IL-6 can effectively identify patients who might have a poor outcome (Fig. 4).

### Predictive accuracy of IL-6 compared with the IMPACT score

**Fig. 5:**
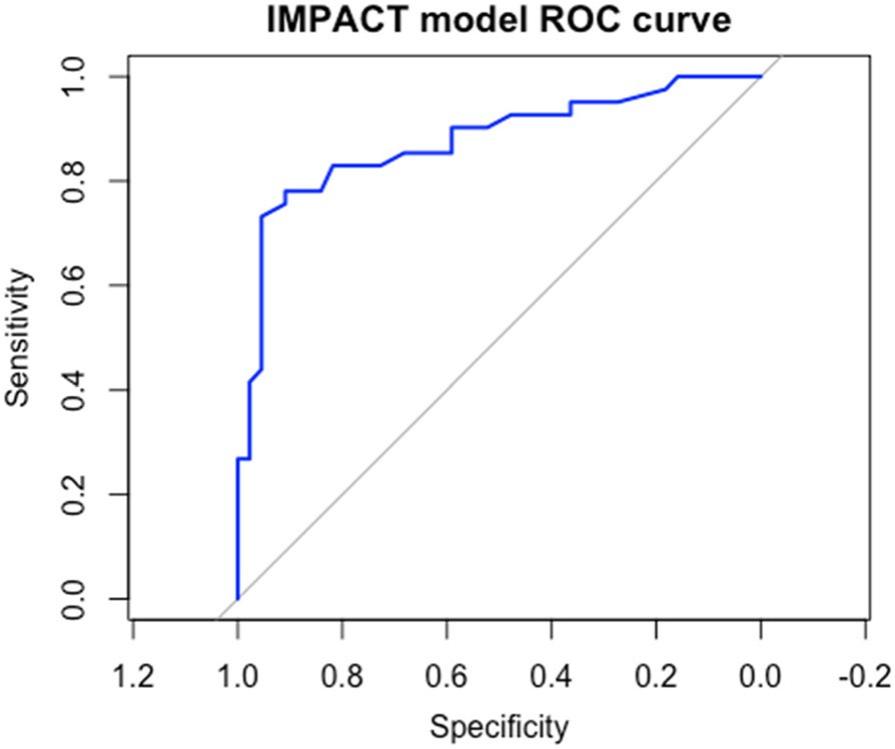
Receiver Operating Characteristic (ROC) Curve for the IMPACT Model Predicting Adverse Outcomes. This ROC curve represents the performance of the IMPACT model in predicting adverse outcomes in traumatic brain injury (TBI) patients. The blue line shows the sensitivity (true positive rate) versus the false positive rate (1-specificity) at various threshold probabilities. The area under the curve (AUC) quantifies the model's overall ability to discriminate between patients with and without adverse outcomes. Here, the IMPACT model achieves a high AUC (0.87 95%CI: 0.79–0.95), indicating strong predictive accuracy. The diagonal gray line represents a random chance reference (AUC = 0.5), emphasizing the IMPACT model's improvement in predictive performance over a non-informative model

In this study, we performed a receiver operating characteristic (ROC) analysis to evaluate the predictive performance of IL-6 levels measured on admission and the established IMPACT score for predicting adverse outcomes in traumatic brain injury (TBI) patients. The area under the ROC curve (AUC) for IL-6 alone was 0.82 (95% CI: 0.73–0.91), suggesting good discriminatory ability for adverse outcomes. In contrast, the AUC for the IMPACT score was 0.87 (95% CI: 0.79–0.95), indicating a higher predictive power compared to IL-6 alone (95% CI 0.03 −0.2, *p* = 0.01) (Fig. 5). However, to formally compare the predictive performance between the IL-6 model (including age and GCS) and the IMPACT score, we applied DeLong’s test, a nonparametric approach for comparing two correlated ROC curves. The test revealed that the difference between the AUC of the IMPACT score and the IL-6 model was not statistically significant (95% CI −0.06—0.08, *p* = 0.7). This suggests that IL-6 has a predictive capacity comparable to the IMPACT score.

We also investigated the impact of integrating IL-6 into the IMPACT model to assess whether this biomarker could improve the model's predictive accuracy. By incorporating IL-6 as an additional variable, the combined model achieved an AUC of 0.89 (95% CI: 0.80–0.96). Although the combined model’s AUC was slightly higher than the original IMPACT score alone, the increase was not statistically significant (95% CI −0.02–0.12, *p* = 0.17), indicating that IL-6 may provide some additional predictive information but does not substantially enhance the overall performance when added to the IMPACT model.

Furthermore, we examined model calibration using the Hosmer–Lemeshow goodness-of-fit test. The combined model, including IL-6, showed poorer calibration compared to the original IMPACT model (Hosmer–Lemeshow *p* = 0.0002), suggesting that adding IL-6 might lead to overfitting or potential misalignment with observed outcomes. Overall, while IL-6 demonstrates potential as a prognostic marker in TBI and offers comparable predictive power to the IMPACT score, its inclusion in the IMPACT model did not significantly enhance performance and may compromise model calibration.

### Missing data and validation

Multiple imputation techniques were used to address missing data, and sensitivity analyses were conducted to ensure the robustness of the findings. Bootstrapping was applied for internal validation of the multivariate models and ROC analyses, confirming the stability of the performance metrics (AUC: 80%, sensitivity 79% and specificity 81%).

## Discussion

This prospective cohort largely resembles the epidemiology of the TBI population reported in other regions worldwide with a predominance of young males and road traffic accidents and falls as the main mechanisms of injury. However, variation in epidemiology exists over regions and countries and has evolved over time [49–52]. The severity of injury by GCS was moderate and severe, and most of them required invasive respiratory support in the field or in the emergency department. Clinical variables associated with an adverse outcome were age, admission GCS, Rotterdam score, hospital infection, and levels of IL-6 on admission. The highest IL-6 levels were documented on admission, followed by a subsequent decrease within the first week. Patients who died within 6 months after injury had higher levels of IL-6. In addition, those with an adverse outcome at 6 months had greater levels of IL-6 compared to those who were independent. In terms of clinical variables and outcome, IL-6 levels on day 0 were associated with TBI severity by GCS and Rotterdam score and with the predicted probability of adverse outcome calculated by the IMPACT-lab model. In addition, IL-6 levels on day 0 was associated with a 6-month adverse outcome. As measured by the GCS, injury severity was moderate to severe, with most patients requiring invasive respiratory support in the field or upon arrival in the emergency department. Clinical variables associated with an adverse outcome included age, admission GCS, Rotterdam score, hospital-acquired infection, and IL-6 levels on admission. The highest IL-6 levels were recorded on admission, followed by a decline within the first week. Patients who died within six months post-injury had elevated IL-6 levels. Additionally, those with an adverse outcome at 6 months had higher IL-6 levels compared to those who regained independence. IL-6 levels on day 0 were also associated with TBI severity (by GCS and Rotterdam score) and with the predicted probability of adverse outcomes, as calculated by the IMPACT-lab model, and were associated with the six-month adverse outcome.

In the multivariable analysis, after adjusting for age, severity of TBI, and the presence of hospital infection, admission IL-6 remained significantly associated with the probability of an adverse outcome at 6 months with a sensitivity of 75% and a specificity of 89% for a cut-off point of 59 pg/ml. These findings were confirmed in a LASSO regularization model to avoid overfitting and multicollinearity. In the LASSO model, all the variables were confirmed to have an impact on the outcome. Furthermore, DCA was performed to evaluate the net benefit of the use of IL-6 in the clinical practice when establishing prognosis, making crucial clinical decisions such as escalating therapies, and providing information to families. DCA demonstrates the clinical utility and net benefit of adding IL-6 to the analysis, particularly in cases when the clinician and family are concerned abouta poor outcome [53]. When integrated into the IMPACT model, IL-6 showed potential for augmenting the model’s predictive power, but also revealed some limitations: the combined model’s AUC was 0.89 (95% CI: 0.80–0.96), which, while promising, did not substantially outperform the IMPACT model’s AUC of 0.87 (95% CI 0.79–0.95). This indicates that while IL-6 provides additional information, it does not drastically shift the overall predictive capacity measured by AUC alone. The addition, IL-6 compromised the model’s calibration, as indicated by a poor Hosmer–Lemeshow *p*-value (*P* = 0.0002). This suggests that the integration may lead to misfit, potentially because IL-6, while strongly associated with outcomes, may overlap or interact in complex ways with variables like GCS and other biomarkers of severity.

Our results confirm the role of relevant clinical variables such as age, severity of injury, and imaging studies in the prognosis of the TBI population. Age has been associated with survival and functionality in TBI, with some studies suggesting that it could be related to a lower pre-injury status and higher comorbidities [49, 54]. In addition, age is part of several TBI prognostication models, namely CRASH [11] and IMPACT models [12], due to its association with outcomes post-injury [50]. In terms of severity of the TBI, admission GCS is widely used as a marker of clinical severity of injury. Although it could be obscured in some cases by sedation and toxics, it has been also demonstrated to have an acceptable correlation with mortality and disability in the TBI population even when compared to other scales [55, 56]. A head CT scan is vital for a comprehensive assessment and is used to make crucial decisions such as neurosurgical interventions [57, 58]. CT findings have been correlated with serum biomarkers in TBI that reflect injury severity [59], and the Rotterdam score has been validated and used for the imaging classification of the injury due to its association with clinical outcomes [34, 60, 61]. Another clinical variable that has a complex clinical interaction in TBI is the occurrence of infections [48, 62–64]. An induced state of immune suppression after acute brain injury has been proposed, driven by the central nervous system and influenced by immune, autonomic, and endocrine changes that lead to a disproportionate number of infections [65–67]. Once patients develop an infection like a respiratory infection or surgical site infection, there seems to be an increased risk of worse outcomes [64, 68–70].

The neuroinflammatory response after TBI has been increasingly recognized in preclinical and clinical settings as a major player in the pathophysiology after injury [71, 72]. Furthermore, depending on the type, strength, and duration, the immune response can support healing and recovery or, if dysregulated, lead to chronic complications [73, 74]. Systemic inflammation, evaluated through the expression of inflammatory cytokines like IL-1B, IL-6, IL-8, IL-15, and TNF-a, is upregulated in the acute phase of TBI and impacts clinical outcomes such as mortality [75, 76], the occurrence of infections in the acute and subacute period [62, 70], and functionality [31, 77]. Furthermore, persistent inflammation several months after TBI has been documented, and it is related to impaired cognition [78], sleep disorders [79], and behavioral changes [80].

While some inflammatory markers such as CRP (C-reactive protein) levels are frequently measured in the clinical settings, none of them have been validated and/or standardized for its clinical use as makers of severity or prognosis [31]. CRP was evaluated as part of a panel of serum biomarkers to identify the presence of intracranial lesions in the brain CT in patients who had suffered mild TBI, in fact CRP, type IV collagenase and creatine kinase T type were significantly able to predict injury in the CT [81]. A different study in patients with TBI of any severity demonstrated that CRP is a promising biomarker to determine the presence of intracranial bleeding [82]. hsCRP (high-sensitiviy CRP) is a more specific and sensitive test to measure very low values of CRP; hsCRP has been demonstrated to be a promising prognostic biomarker in TBI, including a significant relation to the severity of TBI by GCS, disability and mortality [77, 83]. Regarding specifically IL-6 and its association with functional outcome, a previous study found a good performance of IL-6 levels < 24 h post-injury to discriminate adverse outcomes (GOSE ≤ 4) at 3 months after TBI with a sensitivity and specificity of 87% and 95% respectively for a cut-off point of 46 pg/ml [84]; a significant proportion of cases were mild TBI. IL-6 has also been associated with brain death severity of the injury (GCS 3–12 vs GCS 13–15) and findings on the head CT (positive vs. negative) [31]. A different study in TBI patients who went the operative room upon admission found that cases with post-operative IL-6 levels higher than 100 pg/ml had worse outcomes at 3 months [85]. On the contrary, other studies have failed to find a significant association between IL-6 and intracranial pressure [86], mortality or functionality [87]. However these were smaller studies with samples of less than 40 patients.

Our results add valuable information to the available data on the role of inflammation in the TBI population and its use in the clinical setting. IL-6 levels within the first 24 h post-injury are significantly predictive of adverse outcomes in TBI patients. ROC Analysis: Day 0 IL-6 levels demonstrated a strong discriminatory ability with an AUC of 0.89 (95% CI 0.82–0.96) for predicting adverse outcomes, with an optimal threshold at 59 pg/ml. This threshold provided a sensitivity of 78% and specificity of 81%, alongside positive and negative predictive values of 79% and 81%, respectively. DCA indicated a meaningful net clinical benefit for IL-6 when used to predict poor prognosis in addition to individual variables such as age and severity of injury, especially at threshold probabilities relevant for clinical decision-making. This suggests that IL-6 can effectively help identify patients likely to have poorer outcomes. The findings from our ROC analysis also highlight the potential utility of IL-6 as a prognostic biomarker in TBI and underscore some limitations regarding its integration into established clinical prediction models like the IMPACT score. IL-6 levels measured on admission demonstrated a notable ability to discriminate between favorable and adverse outcomes, with an AUC of 0.89, indicating its strong predictive power. Comparatively, the IMPACT score, a validated tool for outcome prediction in TBI, showed an AUC of 0.87, suggesting that it remains a robust model for clinical prognosis. However, the lack of statistically significant differences in AUC between a model including IL-6, age, and GCS versus the IMPACT score, as assessed by DeLong’s test, suggests that IL-6 can independently approximate the predictive performance of the IMPACT score. Determining IL-6 levels on admission offers valuable prognostic insight into TBI, as elevated IL-6 is associated with a higher likelihood of adverse outcomes, making it a potentially useful biomarker for early risk stratification and guiding clinical decision-making.

When IL-6 was incorporated into the IMPACT model, the combined AUC marginally improved to 0.89, but this increase was not statistically significant. This result implies that while IL-6 provides additional information, it may overlap with or fail to add substantial value beyond the existing variables in the IMPACT model. Additionally, the decrease in calibration, as indicated by the poor Hosmer–Lemeshow test result (*p* = 0.0002), raises concerns about potential overfitting and misalignment when IL-6 is added. This misfit may stem from complex interactions between IL-6 and other predictors in the IMPACT model, such as the Glasgow Coma Scale (GCS) and imaging findings, which also reflect injury severity and inflammatory responses.

Compared to the traditional IMPACT score, IL-6 determination provides a complementary prognostic value in TBI. While the IMPACT score remains a robust predictor based on clinical and imaging variables, IL-6 adds a dimension of real-time biological insight into an inflammatory response. This can enhance early risk stratification, particularly in cases where inflammation may play a pivotal role in outcomes, although IL-6 alone does not significantly outperform the IMPACT score. Integrating IL-6 with the IMPACT may not significantly enhance predictive accuracy and may, in fact, compromise calibration. This finding highlights the need for further research to refine the role of IL-6 in prognostic models, potentially through more sophisticated modeling techniques that account for biomarker interactions or through the development of complementary biomarker panels that provide additive predictive value.

### Limitations

This study has several limitations that should be considered when interpreting the findings. First, as a single-center study, the results may have limited generalizability to broader populations. While our cohort was well-characterized in demographics, injury severity, mechanisms of injury, and clinical outcomes, variations in patient management across centers could influence IL-6 levels and outcomes in other settings.

The cohort size is relatively small, which may reduce the statistical power to detect associations and increase the risk of type II errors, particularly when examining subgroup analyses or secondary outcomes. The small sample size also limits our ability to stratify patients by factors such as comorbidities or detailed injury patterns, which could further refine the predictive value of IL-6.

Another limitation is that blood samples for days 3 and 7 were not available for all patients due to discharges or deaths prior to these time points. While IL-6 measurements on admission were available for the entire cohort, missing data at later time points may limit insights into the temporal trends of IL-6 levels in patients with varying clinical trajectories. This gap could also affect the assessment of IL-6’s predictive utility over time, which may differ in patients with early versus delayed outcomes. In addition, we do not have IL-6 measurements after discharge or other markers that may reveal persistent or local inflammation within the brain. We used the relevant association of IL-6 upon admission with the severity of injury and outcomes such as surrogate of the inflammatory response. However additional research is needed to confirm these findings. Additionally, the study lacks external validation of the findings. Future studies should aim to replicate these findings in diverse populations to ensure that the predictive value of IL-6 remains consistent across different clinical contexts. Lastly, IL-6 levels may be influenced by other factors, such as underlying inflammatory conditions or other injuries; this may introduce confusion, affecting the interpretation of IL-6 as a biomarker specific to traumatic brain injury outcomes. In that sense, brain specific biomarkers could be more suited for this role, actually some of them such as GFAP and UCH-L1 have been already implemented in the clinical setting. Nevertheless, by using IL-6 we want to convey the idea that inflammation plays a crucial role in the pathophysiology of TBI and might contribute to its outcome.

These limitations underscore the need for larger, multicenter studies with serial sampling and robust adjustment for potential confounders to more definitively establish IL-6’s role and clinical utility as a prognostic biomarker in traumatic brain injury.

## Conclusion

This study provides evidence that IL-6 measured on admission holds a promising prognostic value in traumatic brain injury (TBI), correlating with both injury severity and long-term outcomes. While integrating IL-6 into established models like the IMPACT score offers additional predictive insights, our analysis suggests that IL-6 alone may not substantially enhance model accuracy in its current form. Future multicenter studies with larger cohorts and serial biomarker sampling are needed to validate IL-6’s utility across diverse populations and settings.

## Data Availability

Data is provided within the manuscript and available upon request.
